# The Indirect Effect of Death Anxiety on Experienced Meaning in Life *via* Search for Meaning and Prosocial Behavior

**DOI:** 10.3389/fpsyg.2021.673460

**Published:** 2021-05-28

**Authors:** Baorui Chang, Jiaxin Cheng, Jiandong Fang, Junhua Dang

**Affiliations:** ^1^Department of Psychology, Faculty of Education, Guangxi Normal University, Guilin, China; ^2^Department of Neuroscience, Faculty of Medicine, Uppsala University, Uppsala, Sweden

**Keywords:** terror management theory, experienced meaning, search for meaning, prosocial behavior, death anxiety

## Abstract

This study investigated the relationship between death anxiety and experienced meaning in life. Six hundred and forty-eight Chinese college students were surveyed using the Death Anxiety Scale, the Prosocial Behavior Scale, and the Meaning in Life Scale. The results showed that death anxiety predicted experienced meaning through three pathways: the first one was through search for meaning singly; the second one was through prosocial behavior singly; and the third one was through search for meaning and prosocial behavior serially, which accounted for the highest proportion of the total effect. This study highlights the positive side of death anxiety.

## Introduction

According to the terror management theory, humans can be aware of their own vulnerability and inevitability of death due to their advanced cognitive abilities, which produces overwhelming anxiety and fear (Greenberg et al., 1986). Given natural and manmade disasters are unavoidable in human life, in current age with highly developed network media, news and information related to various prominent deaths can be seen everywhere at any time (Bovero et al., 2020). However, death salience is not always negative, and emergency events can often bring more prosocial behaviors (Lowe and Fothergill, 2003; Bauer et al., 2016). For example, earthquake victims are more likely to participate in voluntary activities, such as blood donation and rescue (Wang and Wu, 2020). What makes researchers curious is why individuals engage in prosocial behavior in the face of death threat despite its emotional burden. In this study, it is believed that death anxiety will lead people to have a strong motivation to seek meaning in life. Because prosocial behavior meets the need for meaning, people are more willing to engage in prosocial behavior, after which they will experience higher sense of meaning in life. In other words, there is a serial indirect effect: Death anxiety leads to higher motivation to seek meaning in life, which leads to more prosocial behaviors and finally higher experienced sense of meaning.

First of all, death anxiety can promote people to seek meaning in life. The shattered assumption proposes that death crushes people's basic assumptions about the world and the self: The world is good, safe, kind, and meaningful (Janoff-bulman, 1992). Instead, death reminds people that the world is unordered, unsafe, unpredictable, and uncontrollable. Therefore, death salience makes people experience the feelings of loss and damage of meaning in life (Burke et al., 2010). According to the deficit correcting hypothesis, the motivation to search for meaning in life stems from the lack of the presence of meaning (Baumeister, 1991). As a result, when facing life threats, individuals have the psychological needs to find the meaning in life (Baumeister, 1991; Juhl and Routledge, 2014). This idea is supported by empirical evidence. For example, based on a series of studies about rape victims, breast cancer patients, and people infected with HIV, Taylor (1983) pointed out that search for meaning is often the first step in the process of adaptation to life-threatening events. Davis and colleagues found that 90% of bereaved college students were immersed in thinking about death and repeatedly searching for the cause of the event (Davis et al., 1995). Two months after 9/11, two-thirds of surveyed American adults reported that they were searching for meaning in life (Updegraff et al., 2008). Laboratory studies also showed that under the condition of mortality salience, individuals tended to search for meaning in life by defending their cultural worldview (Hayes et al., 2010; Pyszczynski et al., 2010).

Second, prosocial behavior is an important source of meaning in life, which can be influenced by various contextual (e.g., rules, norms, and social network, for a recent review, see Simpson and Willer, 2015), emotional (e.g., parental warmth and support, for a recent review, see Spinrad and Gal, 2018), and cognitive factors [e.g., theory of mind, for a recent meta-analysis, see Imuta et al. (2016)]. On the one hand, according to self-determination theory (Deci and Ryan, 2000), prosocial behavior can increase the sense of meaning in life by satisfying the basic psychological needs. For example, a person can decide to participate in volunteer activities autonomously and realize he/she has the ability to help others and establish relationships with others (Baumeister and Vohs, 2002; Hoffman et al., 2020). On the other hand, because cultural worldview is a fundamental source of meaning and prosocial behavior is generally praised and encouraged by most cultural worldviews, it is natural to expect that prosocial behavior can bring about a sense of meaning for people (Klein, 2017). Consistent with this, Van Tongeren and colleagues found that prosocial behavior had a strong predictive effect on the sense of meaning no matter prosocial behavior was measured by questionnaires or manipulated in experiments (Van Tongeren et al., 2016). Results from the experience sampling method also provides supporting evidence that prosocial behavior can enhance people's experienced sense of meaning (Hofmann et al., 2014). Therefore, prosocial behavior is a major source of obtaining meaning in life (Wong, 1998; Van Tongeren et al., 2016).

Therefore, based on past experimental work that death reminders inspire search for meaning and nonexperimental findings that engaging in prosocial behavior is a robust source of meaning, we predict that death anxiety will be associated with prosocial behavior through search for meaning and prosocial behavior will in turn corresponds with higher sense of meaning.

## Methods

### Participants and Procedures

The study was reviewed and approved by the Department of Psychology, Faculty of Education, Guangxi Normal University. Participants provided their informed consent to participate in this study. Standard biosecurity and institutional safety procedures have been adhered to.

In total, 680 college students were randomly selected for an online survey, in which 32 were excluded due to invalid responses, yielding an efficiency rate of 95.29%. Among the 648 remaining participants, 329 were males and 319 were females. The mean age was 22.26 years (SD = 4.10). Three hundred and fifty-five participants were from the rural area and 293 were from the urban area.

### Measures

#### Death Anxiety

The Chinese version of the Death Anxiety Scale (Templer, 1970) was used to measure death anxiety (e.g., “I am very afraid of death,” “Seeing the dead body makes my hair stand on end”). The original scale consists of 15 items, of which six is reverse scored. Because reverse-scored items often elicit less accurate responses and unintended consequences for the scale structure (Kulas et al., 2019), we only selected the nine forward-scored items. Participants responded using a 5-point Likert scale ranging from 1 (completely disagree) to 5 (completely agree). The items showed a single-factor structure and yielded high internal consistency (Cronbach'α = 0.82). The mean score was adopted, with higher scores indicating higher death anxiety.

#### Prosocial Behavior

The 15-item Prosocial Behavior Scale (Yang et al., 2016) was used to measure prosocial behavior (e.g., “I would like to do something for the class,” “I would like to donate money and materials to the disaster area”). This scale used a 7-point Likert scale ranging from 1 (completely disagree) to 7 (completely agree). The mean score was adopted, with higher scores indicating higher prosocial behavior. The Cronbach's α of the scale in this study was 0.94.

#### Meaning in Life

The Chinese version of the Meaning in Life Scale (Steger et al., 2006; Wang et al., 2016) was adopted. Five items were used to measure experienced meaning (e.g., “I have found a purpose for my life to satisfy myself”), and the other five to measure search for meaning (e.g., “I am searching for meaning in my life.”). Participants responded using a 7-point Likert scale ranging from 1 (completely disagree) to 7 (completely agree). In this study, Cronbach's α of the experienced meaning scale was 0.72, and Cronbach's α of the search for meaning scale was 0.88.

#### Subjective Socioeconomic Status

Participants were shown a ladder indicating different levels of socioeconomic status (1 = the lowest level; 10 = the highest level). They were asked to indicate on which level they stand.

### Data Analyses

SPSS 25.0 software was used for correlation analysis. PROCESS 3.3 (Model 6) was used to conduct the analysis for the serial indirect effect.

## Results

### Correlation Analysis

The results of correlation analysis are shown in Table 1. Death anxiety, search for meaning, prosocial behavior, and presence of meaning were significantly correlated with each other. Gender and subjective socioeconomic status were significantly correlated with experienced meaning, therefore it is necessary to control these two demographic variables in subsequent test of the indirect effect.

**Table 1:** Descriptive statistics and Pearson's correlations for study variables.

**Variables**	***M***	**SD**	**1**	**2**	**3**	**4**	**5**	**6**	**7**
1. Age	22.260	4.102	1						
2. Gender	1.490	0.500	0.006	1					
3. SSS	4.720	1.874	0.012	0.113^**^	1				
4. DA	30.891	6.683	0.074	0.092^*^	0.006	1			
5. SFM	27.073	4.947	0.039	0.061	0.136^**^	0.359^**^	1		
6. PB	82.009	13.646	0.052	0.062	0.183^**^	0.322^**^	0.713^**^	1	
7. EM	23.622	5.116	0.075	0.132^**^	0.196^**^	0.221^**^	0.512^**^	0.607^**^	1

** *p < 0.01*,

* *p < 0.05; SSS, subjective socioeconomic status; DA, death anxiety; SFM, search for meaning; PB, prosocial behavior; EM, experienced meaning*.

### Serial Indirect Effect

There were significant correlations among death anxiety, search for meaning, prosocial behavior, and experienced meaning, which met the statistical requirements for further analysis of the indirect effect of search for meaning and prosocial behavior. After controlling for gender and subjective socioeconomic status, we analyzed the indirect effect of search for meaning and prosocial behavior on the relationship between death anxiety and experienced meaning. The regression analysis results (see Table 2) showed that death anxiety had a significant positive predictive effect on experienced meaning (β = 0.207, SE = 0.037, 95% CI [0.133, 0.280]). When death anxiety, search for meaning, and prosocial behavior were included into the regression equation together, the predictive effect of death anxiety on experienced meaning became insignificant (β = 0.003, SE = 0.033, 95% CI [−0.062, 0.068]). Death anxiety directly predicted search for meaning (β = 0.354, SE = 0.037, 95% CI [0.283, 0.426]) and prosocial behavior (β = 0.079, SE = 0.029, 95% CI [0.021, 0.136]). Search for meaning positively predicted prosocial behavior (β = 0.670, SE = 0.030, 95% CI [0.612, 0.728]). Both search for meaning (β = 0.154, SE = 0.044, 95% CI [0.067, 0.242]) and prosocial behavior (β = 0.472, SE = 0.044, 95% CI [0.385, 0.558]) positively predicted experienced meaning.

**Table 2:** Regression analysis of variables in the model.

**Variables**	**Equation 1 (EM)**		**Equation 2 (SFM)**		**Equation 3 (PB)**		**Equation 4 (EM)**	
	***β***	***t***	***β***	***t***	***β***	***t***	***β***	***t***
Gender	−0.137^**^	−3.624	−0.044	−1.206	−0.024	−0.875	−0.104^**^	−3.369
SSS	0.210^**^	5.600	0.139	3.793	0.095^**^	3.405	0.100^**^	3.194
DA	0.207^**^	5.524	0.354^**^	9.704	0.079^**^	2.683	0.003	0.093
SFM					0.670^**^	22.674	0.154^**^	3.472
PB							0.472^**^	10.662
*R*^2^	0.105		0.149		0.522		0.400	
*F*	25.155^**^		37.560^**^		175.477^**^		85.449^**^	

** *p < 0.01*;

* *p < 0.05; SSS, subjective socioeconomic status; DA, death anxiety; SFM, search for meaning; PB, prosocial behavior; EM, experienced meaning. All variables in the model have been standardized*.

**Figure 1:**
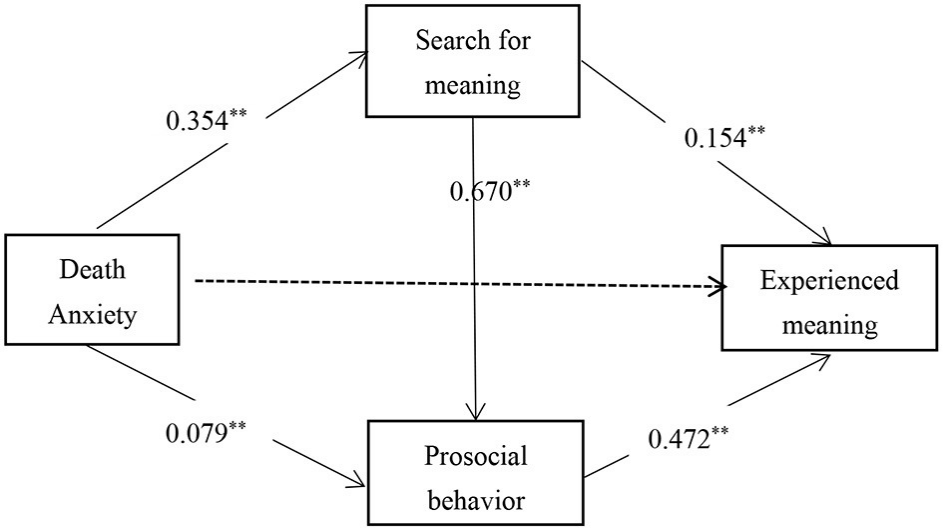
Path diagram of the serial indirect effect. **p < 0.01; *p < 0.05.

The indirect effect analysis showed that search for meaning and prosocial behavior fully accounted for the relationship between death anxiety and experienced meaning, with a total indirect effect value of 0.204. Specifically, the indirect effect was composed of three indirect paths: the indirect effect 1 (Effect = 0.055) from the path of death anxiety → search for meaning → experienced meaning; the indirect effect 2 (Effect = 0.037) from the path of death anxiety → prosocial behavior → experienced meaning; and the indirect Effect 3 (Effect = 0.112) from the path of death anxiety → search for meaning → prosocial behavior → experienced meaning. As shown in Table 3, the three indirect effects accounted for 26.58%, 17.88%, and 54.13% of the total effect, respectively. The bootstrap 95% confidence intervals of the above indirect effects did not contain zero, indicating that the three indirect effects all reached the significant level. There were also significant differences between these three indirect effects. The bootstrap 95% confidence intervals of the difference between the indirect effect 1 and the indirect effect 2 (comparison 1) and the difference between the indirect effect 1 and the indirect effect 3 (comparison 2) contained zero, indicating that there was neither significant difference between the indirect effect 1 and the indirect effect 2, nor between the indirect effect 1 and the indirect effect 3. The bootstrap 95% confidence interval of the difference between the indirect effect 2 and the indirect effect 3 (comparison 3) did not contain zero, indicating that the difference between the indirect effect 2 and the indirect effect 3 was significant. The detailed path model is shown in Figure 1.

**Table 3:** Indirect effect analysis of search for meaning and prosocial behavior.

**Types of indirect effects**	**Effect**	**BootSE**	**BootLLCI**	**BootULCI**	**Relative effect**
Total indirect effect	0.204	0.028	0.149	0.261	98.50%
Indirect effect 1	0.055	0.025	0.008	0.106	26.58%
Indirect effect 2	0.037	0.015	0.009	0.068	17.88%
Indirect effect 3	0.112	0.017	0.080	0.1480	54.13%
Comparison 1	0.018	0.033	−0.047	0.081	
Comparison 2	0.057	0.035	−0.127	0.011	
Comparison 3	0.075	0.019	−0.115	−0.039	

*All variables in the model have been standardized*.

## Discussion

This study examined the relationship between death anxiety and experienced meaning in life and explored its potential mechanisms. Indirect effect analysis showed that search for meaning and prosocial behavior fully accounted for the relationship between death anxiety and experienced meaning. Furthermore, the indirect effect included three paths: The indirect effect of search for meaning, the indirect effect of prosocial behavior, as well as the serial indirect effect of search for meaning and prosocial behavior. This study provided a preliminary answer to the question about how people maintain presence of meaning when they experience death anxiety and why death anxiety increases prosocial behavior.

### Indirect Effects

Death anxiety can predict experienced meaning through search for meaning singly. Steger and colleagues suggested two paths regarding the sequential relationship between search for meaning and experienced meaning: search to presence and the presence to search (Steger et al., 2008). The results of this study are more in line with the search to presence path, namely, heightened motivation to search for meaning leads to higher sense of experienced meaning. A recent longitudinal study also found similar results, such that search for meaning predicted within-person increases in experienced meaning at a later time (Newman et al., 2018).

Death anxiety can also predict experienced meaning through prosocial behavior singly, which is consistent with the terror management theory. That is, in order to alleviate death anxiety, people need to get a sense of value in the cultural context to which they belong and think of themselves as a meaningful and valuable member of the culture (Rosenblatt et al., 1989). Given prosocial behavior is generally valued by most cultures, people are prone to exhibiting prosocial behavior because it can be considered as a way of supporting one's own cultural worldview. Accordingly, ample studies have demonstrated death anxiety could increase prosocial behavior. For example, laboratory studies showed that launching mortality salience promoted charitable behavior (Joireman and Duell, 2007; Chen et al., 2020). Field studies found that participants passing through funeral parlors had a more positive attitude toward charitable organizations (Jonas et al., 2002).

Importantly, the current study found that search for meaning and prosocial behavior serially accounted for the link between death anxiety and experienced meaning. In the first step of this process, death anxiety positively predicts search for meaning, which is in line with the meaning maintenance model (Heine et al., 2006). That is, human beings are creatures that create meaning and seek meaning, and the pursuit of meaning in life is the best way to alleviate the fear of death (Zhang et al., 2019). Because death destroys the possibility of achieving the basic goals of life and the overall belief about meaning in life (Barnett et al., 2018), after a traumatic event involving death threat people tend to reevaluate the event and construct a new meaning (Park and George, 2013). There are many ways to repair the damage of meaning caused by death anxiety. Prosocial behavior is a prominent way of meaning repairing because it is associated with increased sense of meaning in life, which is referred to as the “lingering aroma effect” of prosocial behavior (Van Tongeren et al., 2016; Klein, 2017). Therefore, through search for meaning, death anxiety predicts prosocial behavior, which in turn is associated with higher experienced meaning.

### Limitation and Prospect

Martin Heidegger, in his book *Being and Time*, puts forward the countdown method of the meaning in life for people to face the issue of unavoidable death: To live toward death. That is, since the day of birth, people are approaching death every minute and every second (Heidegger et al., 2010). This study verified the positive relationship between death anxiety and experienced meaning through a cross-sectional method and provided evidence for the path of death anxiety → search for meaning → prosocial behavior → experienced meaning, which sheds light on how to realize “to live toward death.” Although it adds to the current literature, there are still some limitations. First, the research method is relatively simple. In order to better explore the causal relationship between these variables, future studies can employ the longitudinal study method and/or manipulate death anxiety (e.g., increasing mortality salience) in the lab. For example, the classic open-ended short-answer questions or the pictural and audio materials related to death can be used to launch mortality salience (Burke et al., 2010). Prosocial behavior can also be measured by other methods such as the dictator paradigm or the on-site donation paradigm. Second, some studies found that death anxiety could lead to in-group preference and out-group derogation (Rosenblatt et al., 1989). Therefore, future studies can explore whether there are differences in individuals' prosocial behavior toward in-group and out-group when they experience death anxiety.

## Conclusion

This study adopts the questionnaire survey method to investigate the influence of death anxiety on experienced meaning and its underlying psychological mechanisms. It draws the following conclusions: (1) death anxiety predicts experienced meaning via search for meaning; (2) death anxiety predicts experienced meaning via prosocial behavior; and (3) death anxiety positively predicts experienced meaning through a serial indirect effect of search for meaning and prosocial behavior.

## Data Availability Statement

The original contributions presented in the study are included in the article/Supplementary Material, further inquiries can be directed to the corresponding author.

## Ethics Statement

The studies involving human participants were reviewed and approved by Department of psychology, Faculty of Education, Guangxi Normal University. The patients/participants provided their written informed consent to participate in this study.

## Author Contributions

BC developed the study concept. Testing and data collection were performed by BC, JC, and JF. BC, JC, and JD performed the data analysis and drafted the manuscript. All authors approved the final version of the manuscript for submission.

## Conflict of Interest

The authors declare that the research was conducted in the absence of any commercial or financial relationships that could be construed as a potential conflict of interest.
